# Twin-pulse seeding enables pump-probe capabilities in the EUV to soft X-ray spectrum at synchrotron light sources

**DOI:** 10.1038/s41598-023-32496-6

**Published:** 2023-03-31

**Authors:** X. Yang, G. Penn, L. H. Yu, X. Huang, V. Smaluk, T. Shaftan

**Affiliations:** 1 National Synchrotron Light Source II, Brookhaven National Laboratory, Upton, NY 11973 USA; 2 Lawrence Berkeley National Laboratory, Berkeley, CA 94720 USA; 3 SLAC National Accelerator Laboratory, Menlo Park, CA 94025 USA

**Keywords:** High-harmonic generation, Nonlinear optics, Design, synthesis and processing

## Abstract

Having previously reported that separating the two stages of echo-enabled harmonic generation (EEHG) with one or more bending magnet (BM) sections allows the BMs to serve as the desired source of momentum compaction, here we demonstrate that this arrangement can greatly reduce the total energy modulation required by any 4th generation synchrotron light source, leading to higher repetition rates as well as stronger coherent radiation output power, with significant benefits. Since the EEHG beamline performance is mainly determined by the momentum compaction, beam emittances and beta functions of a storage ring lattice, allowing for different separations between the two stages is a straightforward way to increase the momentum compaction of chicane 1. This also enables pump-probe capabilities in a novel context, where twin-pulse seeding on the same electron bunch would allow two distinct radiation pulses with an adjustable delay in the range of 0.1 to 10 ps. In the twin-pulse seeding scheme, the same electron bunch could undergo modulation from two distinct laser pulses. Later stages would produce independent harmonics in subsequent straight sections. There are two variations of this twin-pulse seeding scheme, supporting different scientific applications. With a common modulation in stage 1, the first option allows simultaneously two independent radiation sources, with a full coverage of the EUV (2.5 to 50 nm) to soft X-ray (1.25 to 2.5 nm) spectrum; for the second option, the same stage 2 undulator could generate two coherent pulses both fitting within the FEL bandwidth, or at distinct harmonics. We present particle tracking simulation studies based on the APS-U lattice, including quantum excitation and radiation damping. These simulations indicate that there is no degradation of the modulated longitudinal phase space even when the two stages are separated by as many as 10 BM sections.

## Introduction

The synchrotron light source (SLS) is a major tool for a wide range of scientific endeavors, in particular because of the high pulse repetition rate it enables. Compared to linac sources, the 4th generation SLSs have several key constraints: (1) long bunch length (at least a few picoseconds); (2) large energy spread; (3) low peak current. Because of these limitations, some form of external seeding via a laser system is required to generate shorter radiation pulses^1,2^. When conventional high-gain harmonic generation (HGHG)^3–6^ is utilized, a great deal of seed laser power is required to generate coherent radiation (CR) at wavelengths significantly below what a conventional laser technology can deliver due to the large energy spread. To obtain a significant prebunching, energy modulation must be comparable to $n \cdot \sigma_{E}$, where $n$ is the harmonic and $\sigma_{E}$ is the energy spread, which currently limits the available repetition rate. There are some other approaches to provide intense coherent extreme-ultraviolet (EUV) radiation in a free-electron laser (FEL), e.g., injection of harmonics generated in gas^7^. Also, angular dispersion enhanced prebunching has been proposed as an effective way to reduce the external laser power particularly when the vertical emittance is sufficiently low^8,9^. However, this scheme imposes constrains on the value of dispersion in a particular region of the storage ring (SR), which requires a significant change of the SR lattice.

Echo-enabled harmonic generation (EEHG) is another scheme that is less sensitive to the energy spread. EEHG utilizes two stages of seeding, which combine to produce a large prebunching with energy modulation comparable to the initial energy spread, even for high harmonics. The initial stage requires a significant momentum compaction; however, it can be set to a specific value still having a reasonable range of tunability. Especially, this scheme is well suited to SR-based FELs, as we have shown that the two stages of EEHG can be placed in two straight sections (SSs) separated by one or more bending magnet (BM) sections, which provide the required momentum compaction^1,2^. Thus, EEHG produces short CR pulses^8–16^ at high repetition rates, is fully compatible with SLSs (no need of any lattice change), as well as allows the accessibility to much higher harmonics toward the soft X-ray spectrum. Regarding an external laser, from harmonic 50 to 200, only 30–40% decrease in the achievable bunching factor is predicted while using a reasonable laser power for seeding. There is a significant prebunching at harmonic 200 and even beyond. The resulting fully coherent ultrafast photon pulses up to soft X-ray wavelength could offer unique opportunities to conduct high resolution phase-contrast spectroscopy on organic materials that are important in medicine, biology, and bio-renewable energy materials^17^. Extending the pump-probe approach to soft X-ray could allow detailed studies of excited-state dynamics in organic molecules or biomolecular structures on a nanosecond to femtosecond time scales.

**Figure 1:**
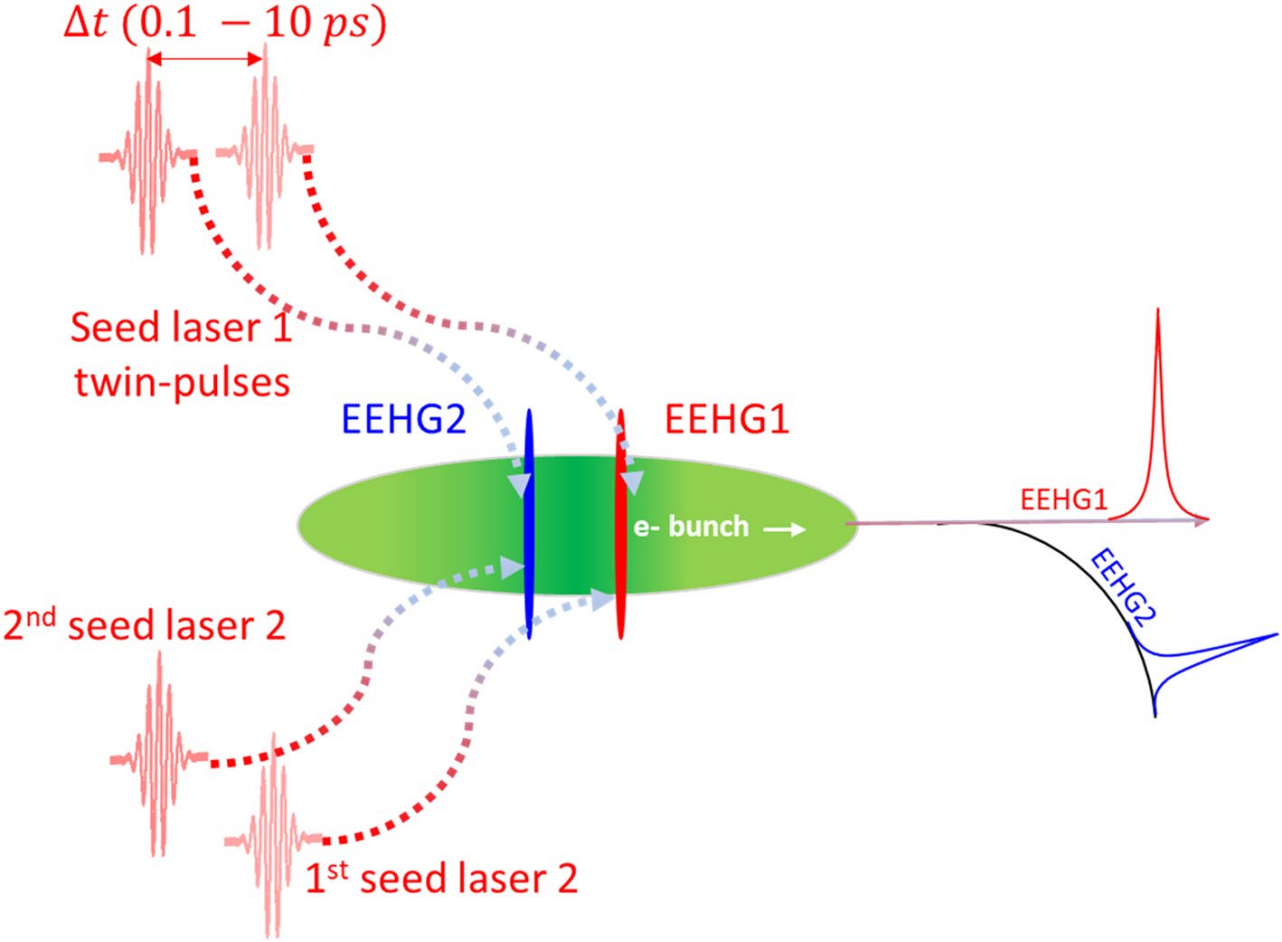
Schematic of the twin-pulse-seed two-color pump-probe experiment.

**Figure 2:**
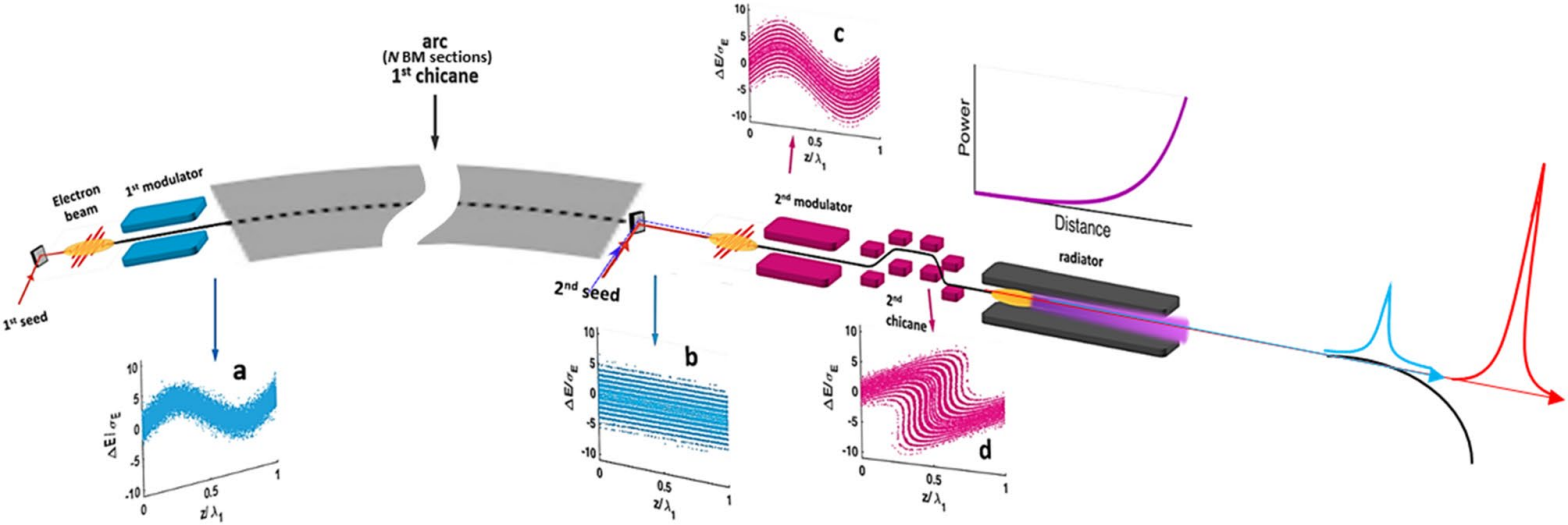
The evolution of the electron energy distribution Δ*E* expressed in terms of its ratio over the energy spread $\sigma_{E}$ along the system length z, expressed in the units of seed laser wavelength $\lambda_{1}$. Panels (**a**–**d**) refer to the first modulator, the first chicane, the second modulator and the second chicane, respectively. The power–distance graph (top right) shows that the radiation is quadratically amplified as it travels through the radiator. Middle: experiment set-up. Harmonic two-color pulses are shown on the right as the red and blue pulses. Figure adapted from Ref.^6^, Springer Nature Limited.

Building on our recent development of utilizing two straight sections (SSs) of a SR to seed coherent emission in the EUV to soft X-ray range^1,2^, we present a modified EEHG, twin-pulse seeding, where multiple laser pulses are used for the first stage of seeding within a single modulator to generate distinct regions of modulation, and the final stage of seeding for each region can be performed in a different SS to allow a broad range of independent tunability. Twin-pulse seeding is fully compatible with the large energy spread of a SR as well as the small momentum compaction per BM section intrinsically associated with a 4th generation SLS, as the desired momentum compaction can be accumulated over multiple BM sections. This technique can produce two or more distinct radiation pulses with adjustable delays in the range of sub-ps to 10 ps, and with wavelengths ranging from 50 nm down to 1.25 nm. This twin-pulse seeding scheme is ideally suited for one to incorporate the pump-probe beamlines into the SR-based light sources. We present two different types of pump-probe beamlines where the initial modulations are produced in a common modulator. The same electron bunch undergoes modulation from two distinct laser pulses. Regarding the first option, later stages could produce two independent harmonics in subsequent SSs, with a full coverage of the EUV (2.5 to 50 nm) to soft X-ray (1.25 to 2.5 nm) spectrum. The schematic of option 1 is shown in Fig. 1 (see details in section "Design of time-resolved two color EEHG beamlines"). For the second option, the same undulator of the later stage could generate two coherent pulses, which both fit within the FEL bandwidth, or at distinct harmonics. The schematic of option 2 (named the harmonic two-color pump-probe beamline) is shown in Fig. 2 (see details in section "Design of a harmonic two-color pump-probe beamline"). Both beamline options offer improved longitudinal coherence, output stability and time-resolved capabilities. Thus, they can potentially broaden the scientific horizon via studying excited-stage dynamics in organic molecules and allowing greater understanding of excited-stage behavior of complex organic molecules^18,19^.

## Results

### Feasibility study of an adjustable two-stage separation

**Figure 3:**
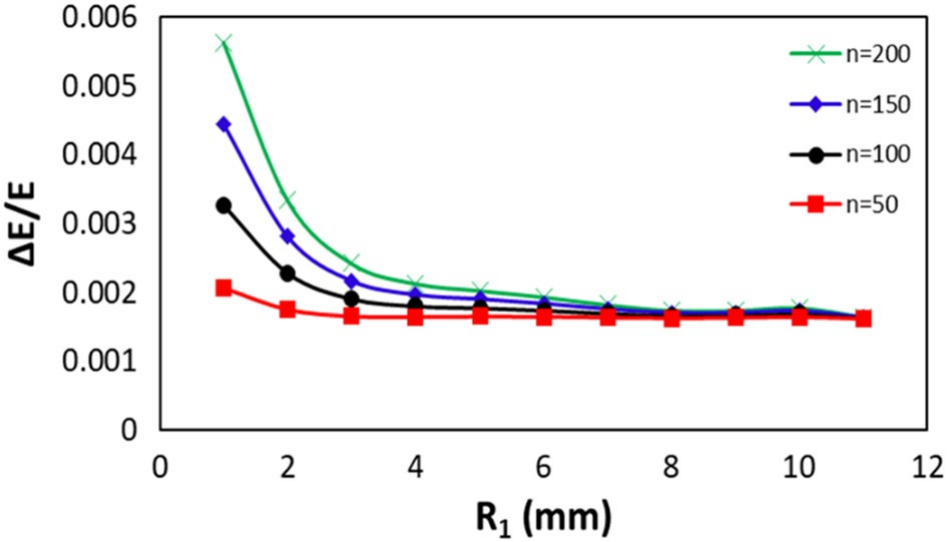
The normalized final energy spread as a function of the momentum compaction of stage 1 is shown for harmonic 50 (red), 100 (black), 150 (blue), and 200 (green), respectively. We assume the relevant electron beam, seed laser and modulator parameters in simulations: the electron beam with the energy 3 GeV, emittances $\varepsilon_{x} = 80 \,{\text{pm}}$ and $\varepsilon_{y} = 8\,{\text{pm}}$, peak current $I_{peak} = 300 \,{\text{A}}$, root-mean-square (RMS) bunch duration 20 ps, and relative energy spread $0.8 \cdot 10^{ - 3}$; the seed laser 1 and 2 with the same wavelength $\lambda_{1,2} = 250\,{\text{nm}}$, and peak powers depending on harmonic numbers below 0.1 GW and 10 GW, respectively; the modulators of stage 1 and 2 with the same period of 20 cm and the same number of periods 5. The peak current can be different for a specific mode in operation or a different SR lattice. In most of the simulations, we set $I_{peak}$ = 300 A, except for the APS-U lattice since the final CR power can be scaled quadratically by the factor $\left( {I_{peak} / 300} \right)^{2}$ ^2^.

Seeding coherent EUV to soft X-ray emission can be accomplished using a compact EEHG design via two SSs of a SR^1,2^. This design utilizes the BM section between these two SSs as the first chicane. However, one of the key EEHG parameters, the momentum compaction of chicane 1, is completely determined by the SR lattice while the energy modulation of stage 1 is chosen to have an optimal and reasonably low value (typically $A_{1} = 2.5$)^1^. For each harmonic, the final energy spread only varies as a function of the momentum compaction of the SR lattice, as shown in Fig. 3. Note that, the resulting slice energy spread becomes larger for a smaller value of the momentum compaction. Compared to the current SLSs, momentum compactions of the future 4th generation diffraction limited SLSs are often a few to ten times smaller, not much larger than 1 mm. Even in a short radiator case (a few meters), the final CR power is negatively correlated to the final energy modulation of the beam slice. Thus, higher final energy spread for the part of the beam that interacts with the external laser leads to lower peak power^2,20,21^.

**Figure 4:**
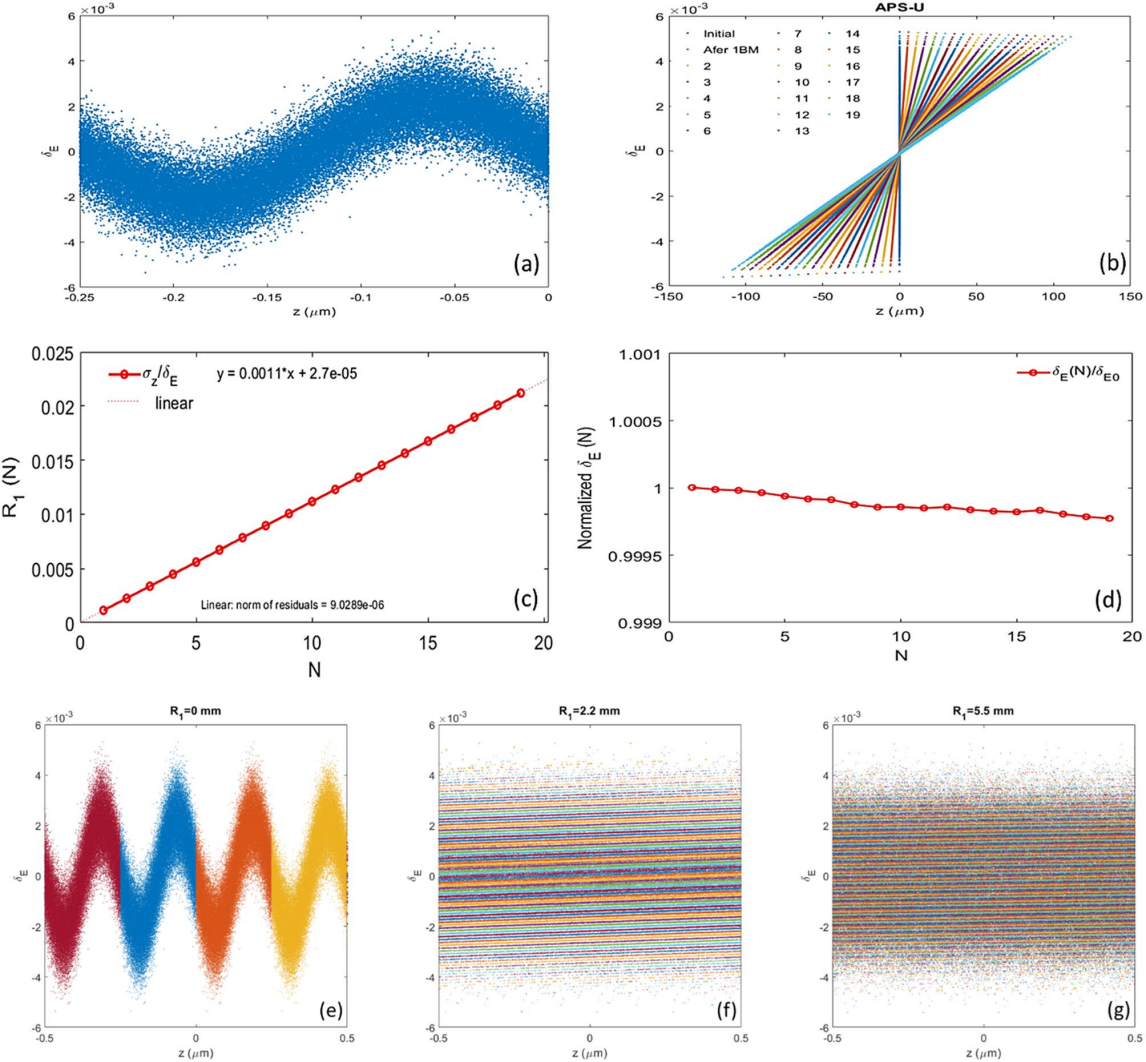
Longitudinal phase space distribution with the slice size of a stage-1 seed-laser wavelength (**a**) right after the stage-1 energy modulation with $A_{1} = 2.5$. (**b**) Longitudinal phase space distributions of the initial, after 1, 2, 3, …, 19 BM sections. (**c**) Momentum compaction, which is extracted from particle tracking simulation via the ratio of the RMS bunch length and the RMS energy spread, as a function of the number of BM sections is plot. (d) RMS energy spread obtained from particle tracking simulation after 1, 2, …, 19 BM sections. Longitudinal phase space distribution in the range of $- 2 \cdot \lambda_{1}$ to $2 \cdot \lambda_{1}$ ($\pm 0.5 \,\upmu {\text{m}}$); (**e**) right after the stage-1 modulation; (**f**) after 2 BM sections; (**g**) after 5 BM sections. The negative z in the plots corresponds to the head of the electron beam. We assume the relevant electron beam, APS-U lattice^26,27^, seed laser and modulator parameters in simulations: the electron beam with the energy 6 GeV, emittances $\varepsilon_{x} = 40\,{\text{pm}}$ and $\varepsilon_{y} = 5\,{\text{pm}}$, peak current $I_{peak} = 200\,{\text{A}}$, RMS bunch duration 50 ps, and relative energy spread $1.3 \cdot 10^{ - 3}$; APS-U lattice with the momentum compaction per BM section $R_{1} = 1.1 \,{\text{mm}}$ and beta functions $\beta_{x} = 5.2\,{\text{m}}$ and $\beta_{y} = 2.4 \,{\text{m}}$; the seed laser 1 with the wavelength $\lambda_{1} = 250 \,{\text{nm}}$, and peak power 280 MW; the modulator of stage 1 with the period of 20 cm and number of periods 5 since we mainly focus on how the longitudinal phase space evolves through various number of BM sections after the stage-1 modulation.

To overcome the small momentum compaction intrinsically associated with any 4th generation SLS^22–24^, separating the stage 1 and 2 with a few more BM sections is considered as an effective way to increase the momentum compaction of chicane 1. To explore how well the longitudinal phase space distribution after the stage-1 energy modulation is preserved after passing through an increased number of BM sections, we perform the following particle-tracking studies^25^ based on the APS-U lattice^26,27^ with $A_{1} = 2.5$. Quantum excitation and radiation damping are turned on in the tracking setup. First, we only track a beam slice with the longitudinal size of one seed laser wavelength ($\lambda_{1} = 250 \,{\text{nm}}$). The longitudinal phase space distribution right after the stage-1 energy modulation is used as the initial distribution (Fig. 4a); then, an equilibrium transverse distribution is added to this longitudinal phase space distribution with a random mixing of the transverse and longitudinal particle index. The longitudinal phase space (δ vs z) of this beam slice is plotted in Fig. 4b after passing through 1, 2, 3, … up to19 BM sections, respectively. Here, δ is the normalized energy deviation, $\delta_{i} = \frac{{E_{i} - E_{0} }}{{E_{0} }}$ for the *i*th particle, and $E_{0}$ is the bunch centroid energy. It is evident that the longitudinal phase space with the stage-1 energy modulation is perfectly preserved after passing through an increased number (*N*) of BM sections, since the ratio of root mean square (RMS) bunch length and the corresponding energy spread is linearly proportional to *N* with the slope as the momentum compaction of one BM section (Fig. 4c) and the normalized energy spread stays nearly constant (Fig. 4d). Also, we track the energy modulated electron beam slice via the stage 1 with a width up to $500 \cdot \lambda_{1}$. So, we can have a longitudinal phase space distribution which is the closest replica of the reality. To have a clear view of the energy stripe formation in the longitudinal phase space, we plot the longitudinal phase space only within the range of $- 2 \cdot \lambda_{1}$ to $2 \cdot \lambda_{1}$ ($\pm 0.5 \,\upmu {\text{m}}$) at the entrance of the first BM section, after 2 BM and 5 BM sections as Fig. 4e–g, respectively.

We also compare the longitudinal phase space evolving through various BM sections via the particle tracking simulation with the calculated distribution via the analytical formula^10,11^, and they are identical.

### Demonstration of power reduction in the second seed laser

**Figure 5:**
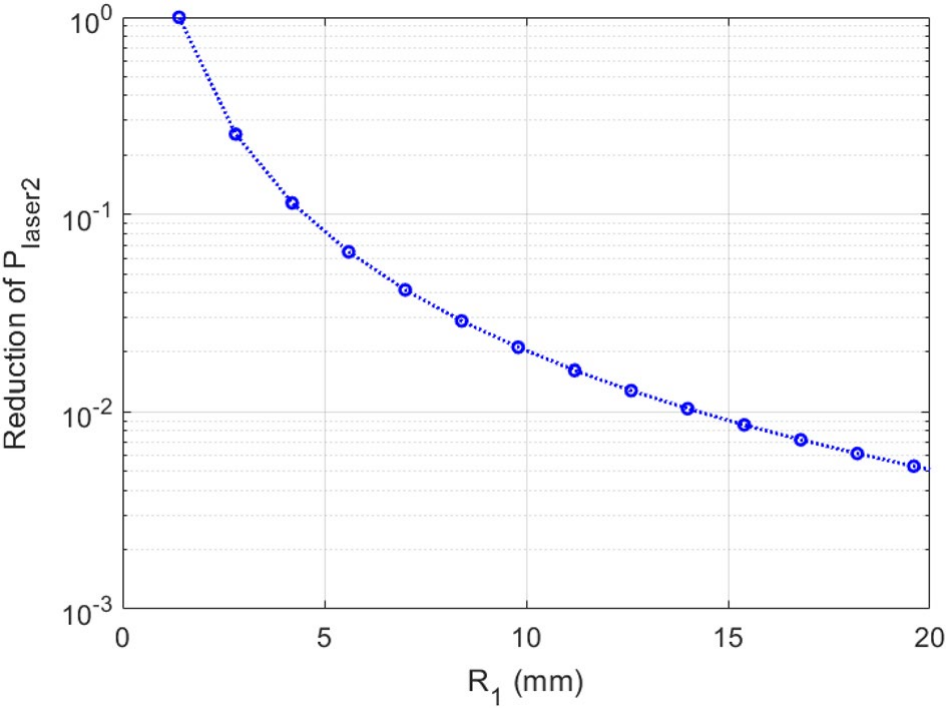
The normalized stage-2 seed-laser power regarding the value with $R_{1} =$ $1 \,{\text{mm}}$ is plotted as a function of $R_{1}$. We assume the relevant electron beam, a 4th generation SLS lattice based on the NSLS-II upgrade^23^, seed laser and modulator parameters in simulations: the electron beam with energy 3 GeV, emittances $\varepsilon_{x} = 25 \,{\text{pm}}$ and $\varepsilon_{y} = 8 \,{\text{pm}}$, peak current $I_{peak} = 300\,{\text{A}}$, RMS bunch duration 20 ps, and relative energy spread $0.8 \cdot 10^{ - 3}$; NSLS-II upgrade lattice with the momentum compaction per BM section $R_{1} = 1.4 \,{\text{mm}}$ and beta functions $\beta_{x,y} \approx 1.0 \,{\text{m}}$; the seed laser 1 and 2 with the same wavelength $\lambda_{1,2} = 250 \,{\text{nm}}$, and peak powers of seed-1 730 MW (due to $A_{1}$ being fixed to 2.5) and seed-2 in the range of 16.0 MW (when $R_{1} = 20 \,{\text{mm}}$) to 3.03 GW (when $R_{1} = 1.4 \,{\text{mm}}$); the modulators of stage 1 and 2 with the same period 20 cm and the same number of periods 5.

One main advantage of separating the two stages of the EEHG beamline with extra BM sections is to reduce the external laser power required. While the energy modulation of stage-1 is fixed to the optimal value ($A_{1} = 2.5$)^1,2^, the required seed-laser power in stage-2 is greatly reduced with the increase of $R_{1}$. This can be understood that for a fixed $A_{1}$, more energy stripes each with smaller energy spread could be formed in the longitudinal phase space as the result of increasing $R_{1}$, thus, the required seed-laser power in stage-2 is reduced for a given harmonic. The seed-laser power required in stage-2, scaled to the value required when $R_{1} = 1 \,{\text{mm}}$, is plotted as a function of $R_{1}$ in Fig. 5. A value of $R_{1} = 4 \,{\text{mm}}$ reduces the required stage-2 seed-laser power by a factor of 10, and a value of 15 mm is sufficient to achieve a factor of 100 reduction; this could potentially result in an increase of the repetition rate by a similar amount, since the stage-2 seed-laser power is currently the leading factor that would limit the repetition rate of EEHG to < 1 kHz^1,2,28,29^.

### Optimization of the two-stage separation

#### Harmonic 50

**Figure 6:**
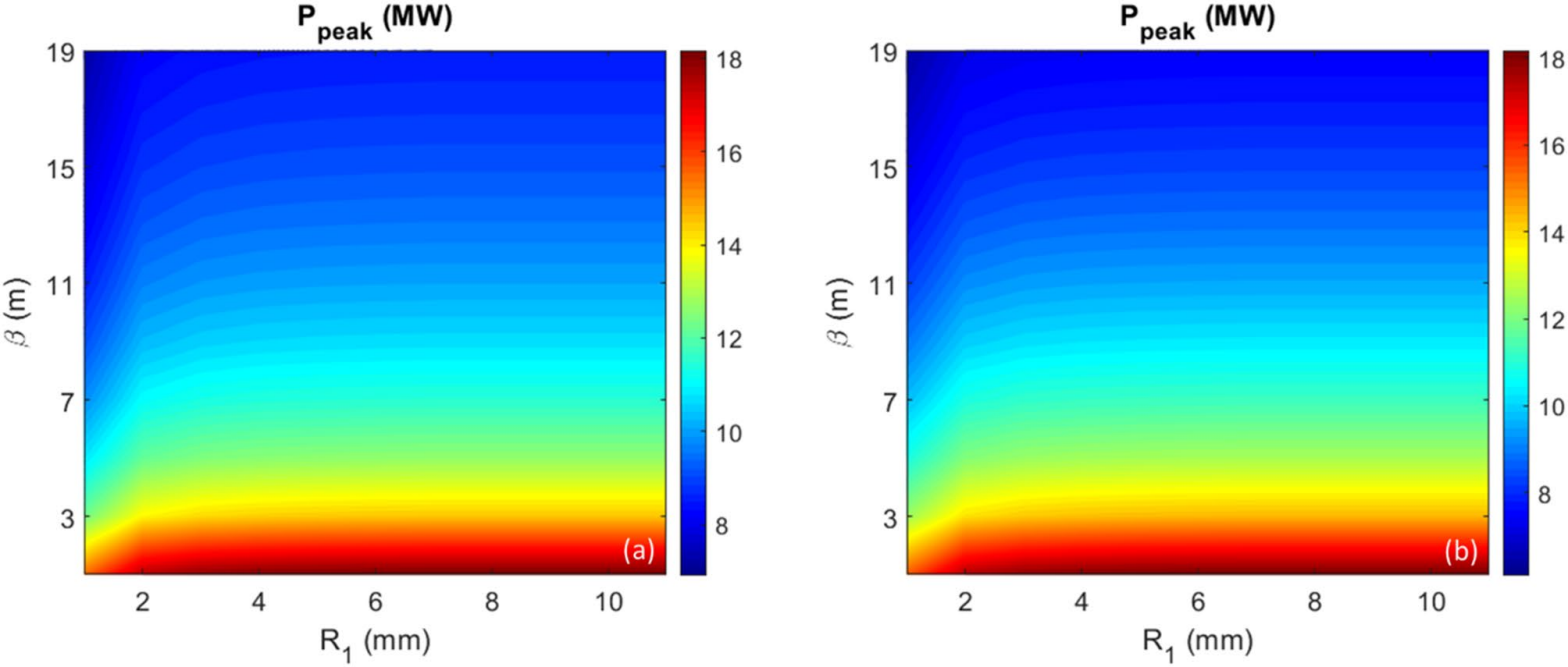
For harmonic 50 and peak current of 300 A, (**a**) Peak power as functions of $\beta$ (y axis) and $R_{1}$ (x axis) is shown as the contour plot calculated by the analytical model. (**b**) Peak power as functions of $\beta$ (y axis) and $R_{1}$ (x axis) is shown as the contour plot obtained by GENESIS simulation.

We study harmonic 50 below as an example of a “low” harmonic. For harmonic 50, the optimal prebunching stays nearly constant (8.5%) despite the variation of the momentum compaction $R_{1}$ in the range 1 to 11 mm. The energy modulation needs to be increased mildly with the decrease of $R_{1}$ (red curve in Fig. 3). This will reduce the CR power at the exit of the radiator with a finite length ($L_{u} = 3.5 \,{\text{m}}$). We scan $R_{1}$ and $\beta$ in the range to cover the complete parameter space while $A_{1}$ and $L_{u}$ are fixed to 2.5 and 3.5 m, respectively. The undulator parameters (period length, number of periods, and *K*-factor) are $\lambda_{u} = 6.4 \,{\text{cm}}$, $N = 55$, and $K_{u,helical} = 2.0946$, respectively. A helical undulator is often used to maximize the CR output power. The peak power ($P_{peak}$) as functions of $\beta$ (y axis) and $R_{1}$ (x axis) obtained by GENESIS^30^ simulation is shown as the contour of Fig. 6b. Similarly, by taking the simulation result $P_{peak}$ as a function of $R_{1}$ when $\beta$ equals to 1 m (see section "Method" for details), $P_{peak}$ as a function of $R_{1}$ with all other values of $\beta$ can be well reproduced for the parameter space with $\beta$ in the range of 1 to 19 m and $R_{1}$ in the range of 1 to 11 mm by an empirical fit of

1 $$ P_{peak} \left( {\beta , R_{1} } \right) = P_{peak} \left( {\beta = 1 m, R_{1} } \right)/\left( {\beta /1 m} \right)^{0.3} . $$

The relative error between those two contours is limited to a few percent, as shown visually in the comparison between Fig. 6a (empirical fit of Eq. 1) and Fig. 6b (data from GENESIS simulations).

**Figure 7:**
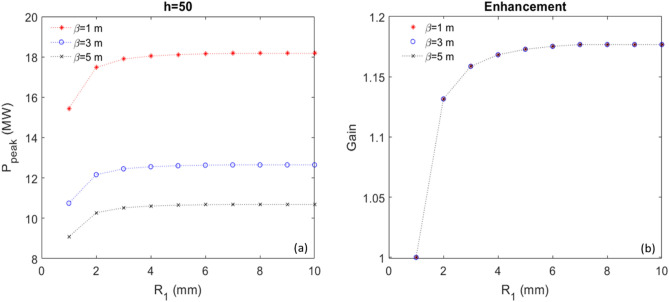
For harmonic 50 and peak current of 300 A, (**a**) Peak power as a function of $R_{1}$ is shown for three cases, $\beta = 1$ m (red), 3 m (blue) and 5 m (black), respectively. (**b**) Normalized peak power, named Gain, regarding the peak power when $R_{1} = 1\,{\text{mm}}$, as a function of $R_{1}$ is shown for three cases, $\beta = 1$ m (red), 3 m (blue) and 5 m (black).

Now, we have an analytical tool which can be applied to study how the CR output power increases with the increase of $R_{1}$, which is linearly proportional to the number of BM sections between those two stages. At the radiator position *Z* = 3.5 m, the peak power as a function of $R_{1}$ is shown in Fig. 7a, comparing three cases with $\beta = 1$ m (red), 3 m (blue) and 5 m (black); the gain of the peak power, which is defined as the normalized value regarding the peak power when $R_{1} = 1 \,{\text{mm}}$, as a function of $R_{1}$ is shown in Fig. 7b. It is evident that the enhancement (gain) due to the increase of $R_{1}$ is independent of the transverse beam size, described by the hybrid parameter scaled $\beta$^1,2^. Regarding the low harmonic, one only can increase the gain by about 16% with the increase of BM sections to 2; afterwards, such gain almost reaches the saturation.

#### Harmonic 200

**Figure 8:**
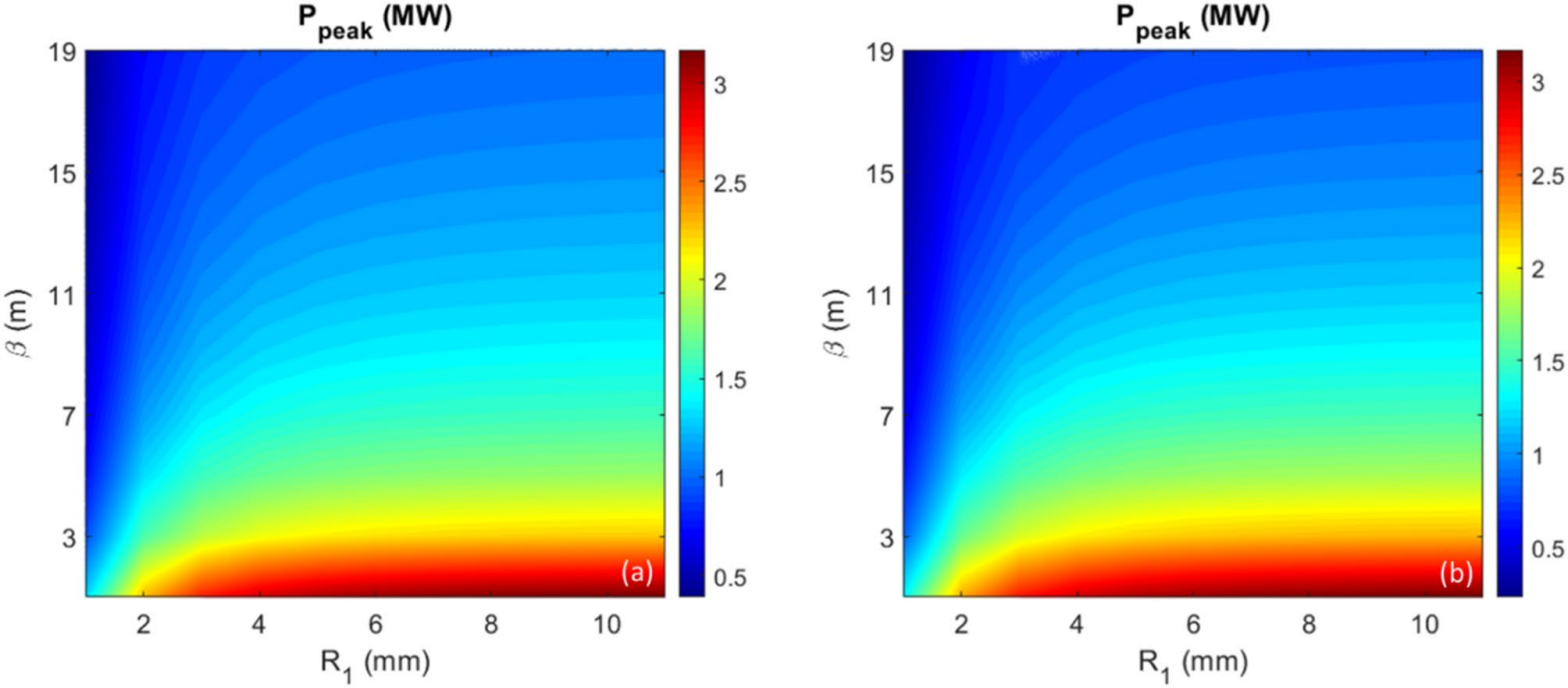
For harmonic 200 and peak current of 300 A, (**a**) Peak power as functions of $\beta$ (y axis) and $R_{1}$ (x axis) is shown as the contour plot calculated by the analytical model. (**b**) Peak power as functions of $\beta$ (y axis) and $R_{1}$ (x axis) is shown as the contour plot obtained by GENESIS simulation.

We study harmonic 200 as a point of comparison for higher harmonics. For harmonic 200, the optimal prebunching still stays nearly constant (5.3%) despite the variation of the momentum compaction $R_{1}$ in the range 1 to 11 mm. The energy modulation needs to be increased significantly (up to 3 times) with the decrease of $R_{1}$ (green curve in Fig. 3). This will reduce the CR power at the exit of the radiator with a finite length ($L_{u} = 3.5 \,{\text{m}}$). We scan $R_{1}$ and $\beta$ in the range to cover the complete parameter space while $A_{1}$ and $L_{u}$ are fixed to 2.5 and 3.5 m, respectively. The undulator parameters (period length, number of periods, and *K*-factor) are $\lambda_{u} = 5.0 \,{\text{cm}}$, $N = 70$, and $K_{u,helical} = 0.8508$, respectively. Also, a helical undulator is used to maximize the CR output power. The peak power ($P_{peak}$) as functions of $\beta$ (y axis) and $R_{1}$ (x axis) obtained by GENESIS simulation is shown as the contour of Fig. 8b. Similarly, by taking the simulation result $P_{peak}$ as a function of $R_{1}$ with other values $\beta$ within the same parameter space as previously considered can be well reproduced by the empirical fit

2 $$ P_{peak} \left( {\beta , R_{1} } \right) = P_{peak} \left( {\beta = 1 m, R_{1} } \right)/\left( {\beta /1 m} \right)^{0.43} . $$

The relative error between those two contours is in the level of 10% or less, and the resulting contour plots are compared in Fig. 8a (empirical fit) and Fig. 8b (GENESIS simulations). The larger exponent for harmonic 200 can be attributed to the reduced importance of diffraction for shorter wavelengths.

**Figure 9:**
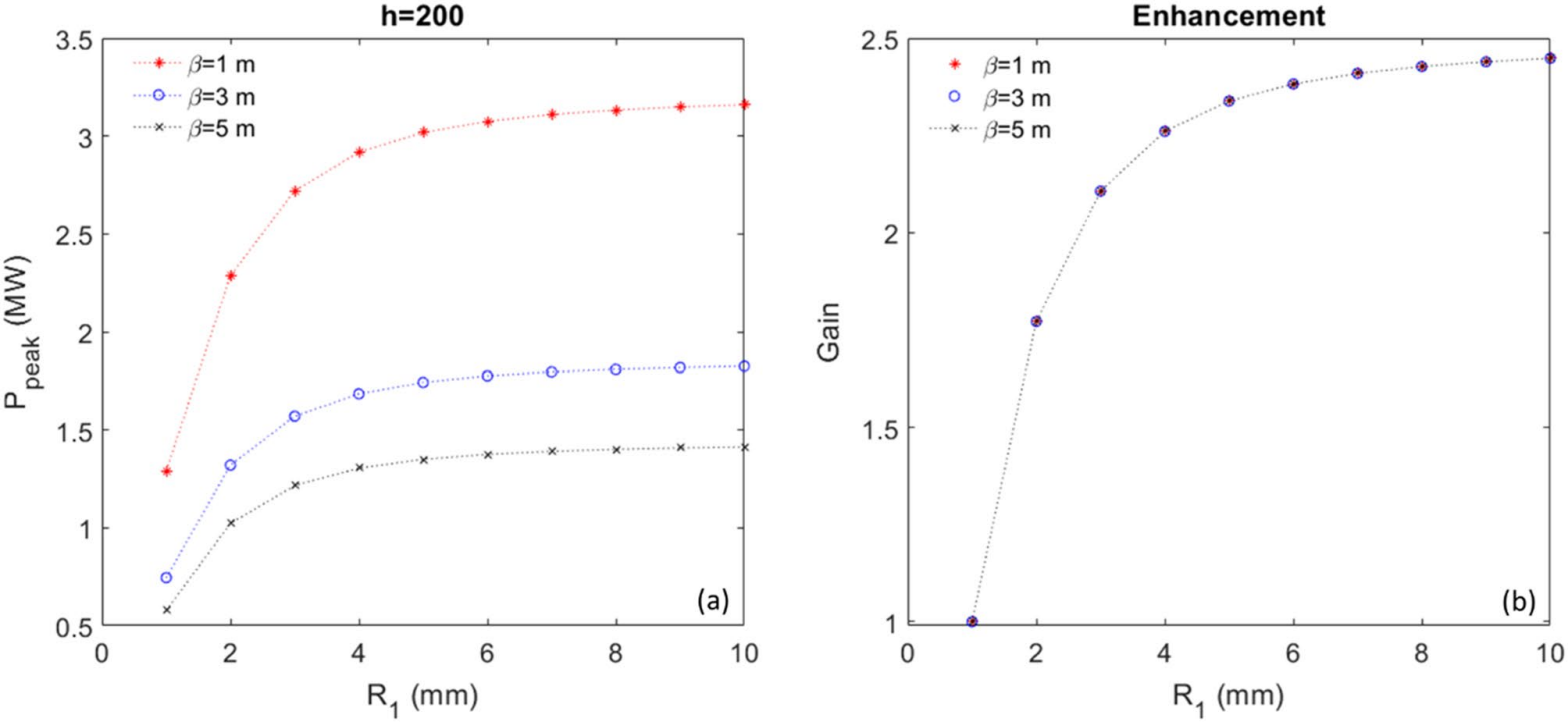
For harmonic 200 and peak current of 300 A, (**a**) Peak power as a function of $R_{1}$ is shown for three cases, $\beta = 1$ m (red), 3 m (blue) and 5 m (black), respectively. (**b**) Normalized peak power, named Gain, regarding the peak power when $R_{1} = 1\,{\text{mm}}$, as a function of $R_{1}$ is shown for three cases, $\beta = 1$ m (red), 3 m (blue) and 5 m (black).

Similarly, we can apply the analytical tool to study how the CR output power increases with the increase of $R_{1}$. At the radiator position *Z* = 3.5 m, the peak power as a function of $R_{1}$ is shown in Fig. 9a, comparing three cases with $\beta = 1$ m (red), 3 m (blue) and 5 m (black); the gain of the peak power as a function of $R_{1}$ is shown in Fig. 9b. It is evident that the enhancement (Gain) due to the increase of $R_{1}$ is independent of the transverse beam size, described by the hybrid parameter scaled $\beta$^1,2^. Regarding the high harmonic, one can significantly increase the gain by a factor of 2 to 3 with the increase of BM sections ≥ 4; afterwards, such gain nearly reaches the saturation.

### Design of time-resolved two color EEHG beamlines

Based on the gain curves of the low (50) (Fig. 7b) and high (200) (Fig. 9b) harmonic examples, for $h = 200$, with the increase of the BM separation to ≥ 4 BM sections, one can gain a factor of 2.5 in the CR output power; instead, for $h \le 100$, stage 2 can be placed as close as 1 BM section downstream of the stage 1. This feature, together with a modest modification of the layout, enables the two-color pump-probe capability via twin-pulse seeding of different locations in the same electron bunch with an adjustable delay between those two pulses in the range of 0.1 to 10 ps. The schematic of this novel approach is shown in Fig. 1. One can modulate the same electron bunch with two different beam slices via stage 1, then, those two beam slices can be modulated independently via two different stage 2. This means that, for a SR serving two EEHG undulators, our method can deliver two different temporally correlated picosecond CR pulses to these two specific EEHG beamlines while leaving the electron bunch going to the other undulators unchanged. Regarding $h \le 100$, the stage 2 can be placed 1 to 3 BM sections downstream of stage 1; it covers EUV spectrum from 2.5 to 50 nm. Instead, regarding $100 < h \le 200$, the stage 2 should be placed at least 4 BM sections downstream of stage 1; it covers soft X-ray spectrum from 1.0 to 2.5 nm. The EUV and soft X-ray EEHG beamlines can be pumped by the same laser that is used to seed the stage 1 modulation, thus, those two beamlines can be timing correlated with each other. The details of the pump probe experiments are beyond the scope of the manuscript since we only focus on the design of the pump-probe beamlines on the accelerator side.

**Figure 10:**
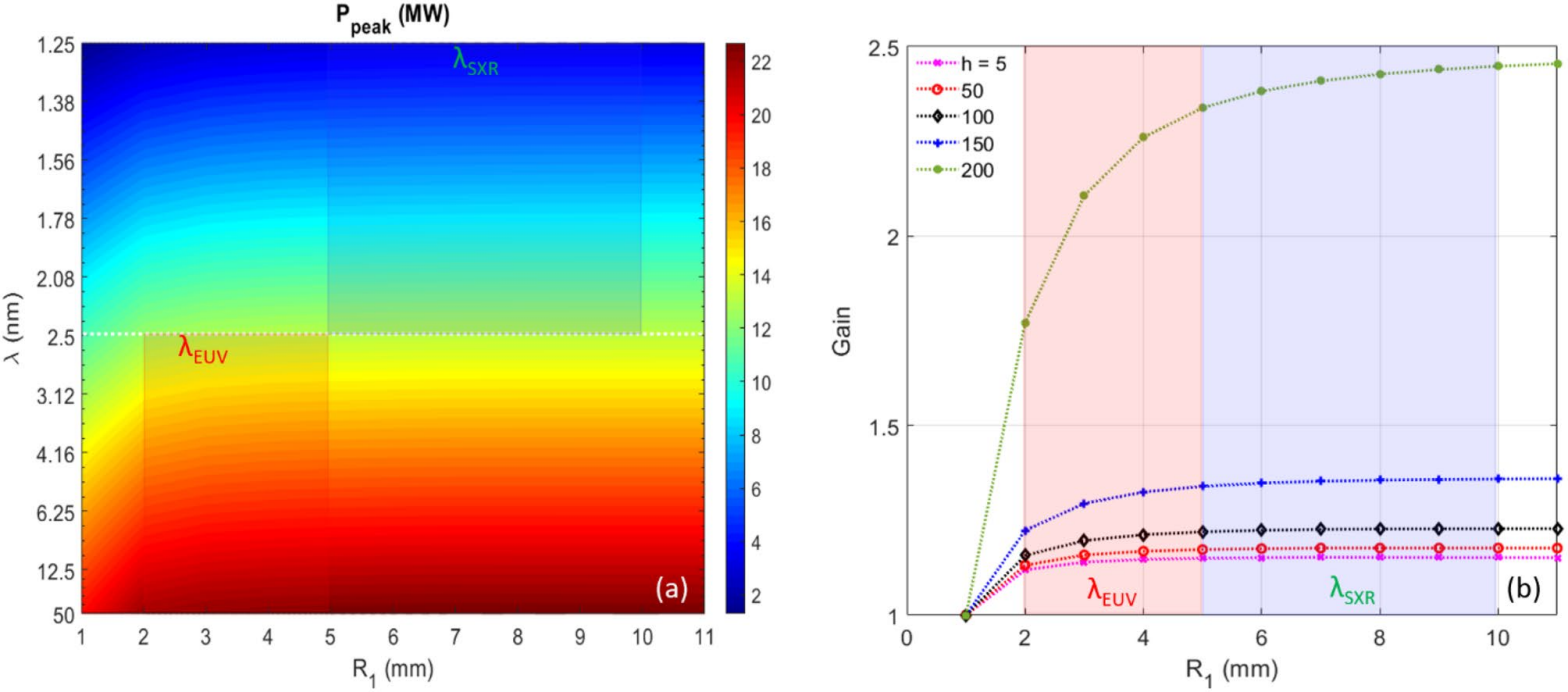
For peak current of 300 A, (**a**) Peak power as functions of $\lambda$ (y axis) and $R_{1}$ (x axis) is shown as the contour plot. The EUV and soft X-ray beamlines are labelled as $\lambda_{EUV}$ and $\lambda_{SXR}$, respectively. (**b**) Gain as a function of $R_{1}$ is plotted for harmonics 5 (magenta), 50 (red), 100 (black), 150 (blue), and 200 (green), respectively. The EUV and soft X-ray beamlines are highlighted as pink and light blue, respectively.

We plan to use the analytical tool described in section "Harmonic 50" and section "Harmonic 200" to calculate the two-color twin-pulse-seed EEHG beamline performances. Since we studied the low (50) and high (200) harmonics in great details and expect other harmonics should be bounded between the results of these two harmonics, we simply apply the linear interpolation method to extend the low and high harmonic model to all harmonics. We calculate $R_{1}$ and $\lambda$ in the range to cover the complete parameter space while $A_{1}$ and $L_{u}$ are fixed to 2.5 and 3.5 m, respectively. The undulator parameters (period length, number of periods, and *K*-factor) are for the EUV beamline $\lambda_{u} = 6.4 \,{\text{cm}}$, $N = 55$, and $K_{u,helical} \approx 1.3014 - 7.2714$, and for the soft X-ray beamline $\lambda_{u} = 5.0 \,{\text{cm}}$, $N = 70$, and $K_{u,helical} \approx 0.8508 - 1.5646$, respectively. The peak power ($P_{peak}$) as functions of the radiation wavelength $\lambda$ (y axis) and stage-1 momentum compaction $R_{1}$ (x axis) is shown as the contour plot in Fig. 10a. The EUV beamline covers $R_{1}$ in the range of 2–5 mm, labelled as $\lambda_{EUV}$; instead, the soft X-ray beamline covers $R_{1}$ in the range of 5–10 mm, labelled as $\lambda_{SXR}$. The gain of CR output power as a function of $R_{1}$ is plotted in Fig. 10b; the EUV and soft X-ray beamlines are highlighted as pink and light blue regimes.

### Design of a harmonic two-color pump-probe beamline

Harmonic two-color pulses can be generated in the same beamline for the pump-probe experiments with the tunability in both the spectrum and the temporal delay. One can develop a harmonic two-color scheme, which allows us to scan the delay between those two pulses in the range of 0.1 to 10 ps. The pulse duration can be varied in the range of tens femtoseconds up to a picosecond. The schematic of the harmonic two-color pump-probe beamline is shown in Fig. 2.

**Figure 11:**
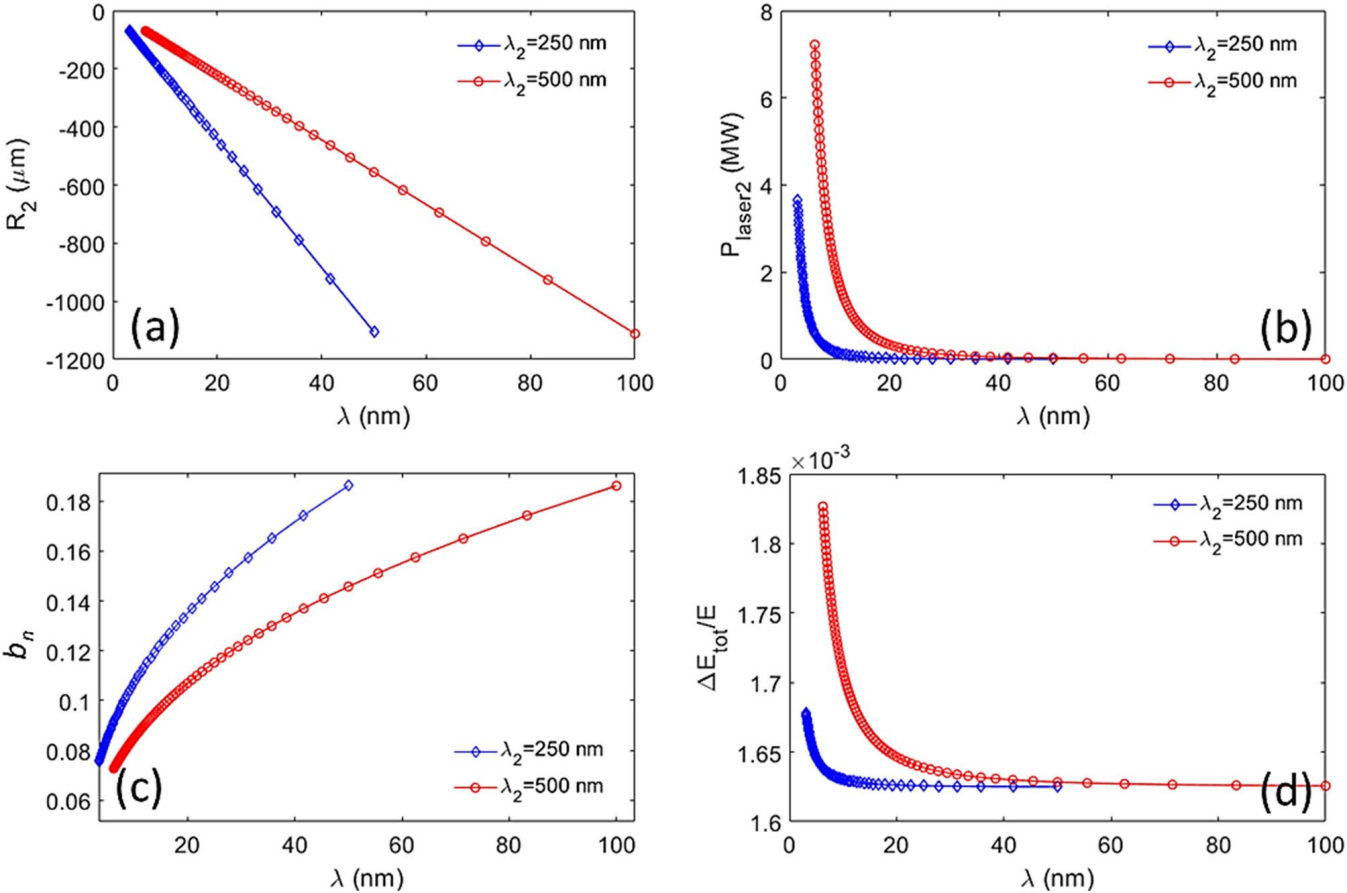
For $A_{1}$ and seed-laser-1 power being fixed to 2.5 and 1.12 MW, regarding the harmonic two-color pulses, (**a**) the momentum compaction of chicane 2, (**b**) seed-laser-2 power, (**c**) bunching factor, and (**d**) normalized final energy spread are plotted respectively.

**Figure 12:**
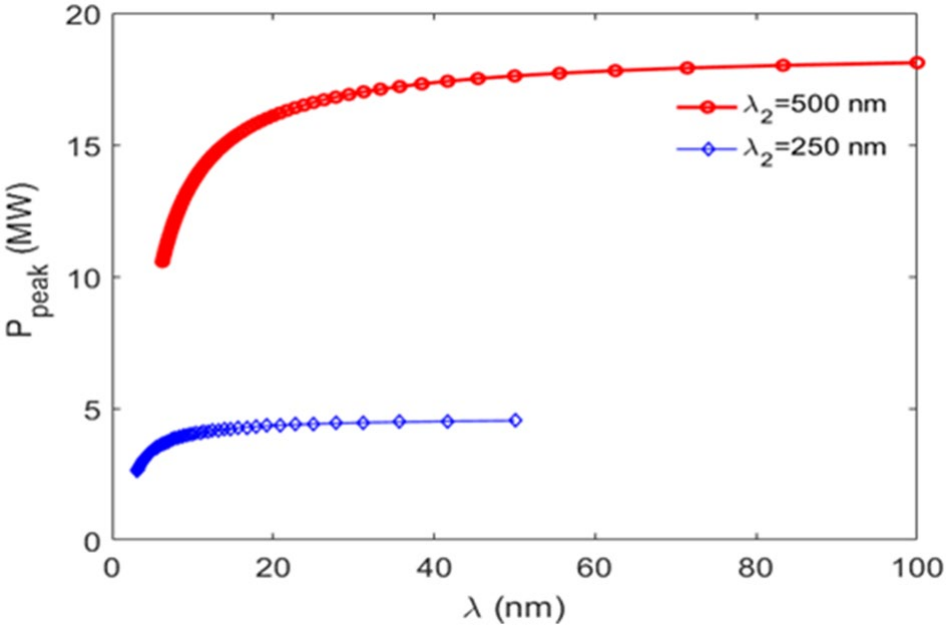
Regarding those harmonic two-color pulses, the peak power as a function of wavelength is plotted for the first (red) and second (blue) pulses, respectively.

However, the design challenges are the harmonic two-color pulses must share the same stage 2, including modulator 2 and chicane 2, and radiator. To achieve a femtosecond-level timing jitter between the pump and the probe, it is necessary that both pulses be generated by the same electron bunch, and the two seed-laser pulses in stage 1 and two seed-laser pulses in stage 2 be generated from the same laser system. We choose both seed-laser-1 pulses with the wavelength of $\lambda_{1} = 500 \,{\text{nm}}$ and those two seed-laser-2 pulses with the wavelength of $\lambda_{2} = 500 \,{\text{nm}}$ and $250 \,{\text{nm}}$, respectively. Thus, to take the harmonic relation of the seed-laser-2 wavelengths into account, these two-color pulses could simultaneously satisfy the fundamental and second harmonic resonance conditions of stage 2, respectively. Their momentum compaction of chicane 2, seed-laser power in stage 2, bunching factor, and normalized final energy spread are shown as Fig. 11 a–d, respectively. The radiator parameters (period length, number of periods, and *K*-factor) are $\lambda_{u} = 6.4 \,{\text{cm}}$, $N = 55$, and $K_{u,helical} = 1.3014 - 10.3318$. The radiator could generate two coherent pulses at distinct harmonics, 2.5 nm to 100 nm for the fundamental and 1.25 nm to 50 nm for the second harmonic, respectively. It is evident that these harmonic two-color pulses can share the same modulator 2, chicane 2 (Fig. 11a), and the radiator, with the similar bunching factors (Fig. 11c). The peak power as a function of wavelength for those harmonic two-color pulses is shown in Fig. 12.

### Estimate of CSR instability for the APS-U lattice

A fundamental aspect of the EEHG process is that the microbunching from the laser modulation is quickly suppressed after it passes through the first chicane with a large momentum compaction $R_{1}$, as the energy modulation folds over on itself multiple times (Fig. 4f,g). Only in the second stage does such impact resurface (named "echo"). We considered the effects of incoherent and coherent synchrotron radiation (ISR and CSR) similar to Xiang and Stupakov^11^. The ISR induced energy spread for four BM sections of the APS-U lattice with $R_{1} = 4.4 \,{\text{mm}}$ is about 50 keV^26,27^. Compared to the separation between adjacent energy bands estimated by $\frac{{\lambda_{1} /2}}{{R_{1} }} \cdot E_{0} = \frac{{250 \,{\text{nm}}/2}}{{4.4\,{\text{mm}}}} \cdot 6 \,{\text{GeV}} = 170 \,{\text{keV}}$ (consistent with the result predicted by the numerical method^11^), one could expect the minor degradation of the bunching factor. In the first chicane, since the beam isn’t micro-bunched, the CSR effect should be negligible; in the second chicane, the beam is briefly micro-bunched only at the last dipole (often $R_{2} \ll R_{1}$). The situation is quite similar to what is being considered by Xiang and Stupakov^11^. Moreover, since the peak current of a SR is significantly lower (e.g., 200 A in APS-U compared to 800 A in linac^11^), the CSR effect should be much weaker.

To have an in-depth understanding of the CSR effect across multiple bending magnets, we estimate the CSR instability for the APS-U lattice. The phenomenon that CSR can drive microbunching instability (MBI) in short electron bunches that traverse bending fields has been studied extensively. Here, we apply the theory developed by Stupakov and Heifets^31^ to estimate the MBI threshold in APS-U SR.

The theory states that for steady-state CSR regarding a ring with a positive phase slippage factor *η*, the beam becomes unstable if the following condition is satisfied,

3 $$ k \cdot r < 2.0 \cdot {\Lambda }^{\frac{3}{2}} $$

where ${\Lambda } = \frac{{n_{b} \cdot r_{0} }}{{\eta \cdot \gamma \cdot \delta_{E}^{2} }}\frac{r}{\langle r\rangle }.$ Here, $k = \frac{2\pi }{\lambda }$ is the mode wave number, $\lambda$ is the wavelength, $r$ is the bending radius, $\langle r\rangle$ is the average ring radius, $n_{b}$ is the linear particle density, $r_{0} = 2.818 \times 10^{ - 15}$ m is the classical electron radius, $\gamma$ is the Lorentz energy factor, and $\delta_{E}$ is the RMS energy spread. The maximum growth rate is reached when the mode wave number $k = 0.68 \cdot {\Lambda }^{\frac{3}{2}} /r$, and can be estimated as $\left( {{\text{Im}}\omega } \right)_{max} = 0.43 \cdot {\Lambda }^{3/2} \cdot c \cdot \eta \cdot \delta_{0} /r$, where $c$ is the speed of light in vacuum.

For APS-U, we have $$\eta = 4.0 \times 10^{ - 5}$$, $$\gamma = 1.174 \times 10^{4}$$, $$\delta_{E} = 1.3 \times 10^{ - 3}$$, $$r = 61$$ m, and $$r = 175.7$$ m. The peak linear particle density parameter ($$n_{b} )$$ is related to the peak current through $$I_{peak} = n_{b} \cdot e \cdot c$$, where $$e = 1.6 \times 10^{ - 19}$$ C is the electron charge. For the peak current of $$I_{peak} = 200$$ A, we have $$n_{b} = 4.2 \times 10^{12}$$/m and $${\Lambda } = 5.21 \times 10^{3}$$. Hence, the mode with the maximum growth rate has the wavelength of $$\lambda_{m} = 1.5$$ mm, and the shortest unstable wavelength is $$\lambda_{0} = 0.51$$ mm.

The electron bunch will experience MBI if the beam can support CSR mode with the wavelength longer than 0.51 mm. However, it is well known that CSR wake fields with long wavelengths are suppressed by the vacuum chamber shielding^32,33^, with the cutoff wavelength given by

4 $$ \lambda_{cutoff} = 2 \cdot b \cdot \sqrt{\frac{b}{r}}  , $$

where $b$ is the half height of the vacuum chamber. For APS-U, $b = 0.012\,{\text{m}}$, hence, $\lambda_{cutoff} = 0.34 \,{\text{mm}}$. As a result, the electron beam is stable with the peak current of $200 \,{\text{A}}$. Based on the above equations, to have an unstable CSR mode shorter than the cutoff wavelength requires the peak current to be above 260 A, significantly above the nominal value.

In the EEHG process considered here, the electron bunch is modulated by a seed laser with wavelength 250 nm. The energy modulation will turn into a density modulation for a brief period in the first BM section, but the density modulation then rapidly folds over on itself multiple times along the z-coordinate and effectively smooths the density modulation, leaving only internal structure hidden in the longitudinal phase space. Furthermore, as shown in the above CSR analysis, the cutoff wavelength for instability is far longer than the seed laser wavelength. This confirms that the CSR effect on the beam should be minor.

## Method

We built a generalized model in our early study, which can predict the EEHG beamline performance for nearly any SLS^2^. The beam emittances and beta functions determine the transverse beam sizes; thus, they will impact the EEHG beamline performance. The transverse beam size needs to be considered. To take most of the 4th generation SLSs into account, we choose the averaged horizontal beam emittance ($80 \,{\text{pm}}$) and beam energy (3 GeV) and assume a 10% coupling for the vertical emittance ($8 \,{\text{pm}}$). The scaling factor, named scaled $\beta$, shown as Eq. (6), is used to map the EEHG performance using Figs. 4 and 5.

5 $$ \beta = \sqrt {\left( {\beta_{x} \cdot \varepsilon_{n,x} } \right) \cdot \left( {\beta_{y} \cdot \varepsilon_{n,y} } \right)/\left( {\varepsilon_{n,x}^{0} \cdot \varepsilon_{n,y}^{0} } \right)} $$

For a specific SLS, $\beta_{x,y}$ are the x and y beta functions; $\varepsilon_{n,x}$ and $\varepsilon_{n,y}$ are the normalized x and y emittances. $\varepsilon_{n,x}^{0} = \gamma_{0} \cdot 80 pm$ and $\varepsilon_{n,y}^{0} = \gamma_{0} \cdot 8 pm$ are the normalized emittances and ($\gamma_{0} = \frac{E}{{m_{e} c^{2} }} = 3000/0.511 \approx 5870.8)$ is the Lorentz factor used in simulations. Here, $\left( {m_{e} c^{2} = 0.511 MeV} \right)$ is the electron rest mass energy. Alternatively, scaled $\beta$ can be converted to the conventional definition of the beam cross section ${\Sigma }$ via Eq. 6.

6 $$ {\Sigma } = \sqrt {\frac{{\beta_{x} \cdot \varepsilon_{n,x} }}{\gamma } \cdot \frac{{\beta_{y} \cdot \varepsilon_{n,y} }}{\gamma }} = \frac{\beta }{\gamma } \cdot \sqrt {\varepsilon_{n,x}^{0} \cdot \varepsilon_{n,y}^{0} } = \frac{\beta }{\gamma } \cdot \left( {1.485 \cdot 10^{ + 5} \cdot {\text{pm}}} \right) $$

The energy modulation needs to be increased substantially with the increase of the harmonic, especially for 4th generation SLSs with small momentum compactions^1,2^. In a short radiator case, the final CR power is negatively correlated to the final energy modulation of the beam slice. To overcome the small momentum compaction intrinsically associated with any 4th generation SLS, we demonstrated that by separating stage 1 and 2 with a few extra BM sections, the momentum compaction of the stage 1 can be significantly increased. The relationship between the momentum compaction of stage 1 ($R_{1}$) and the number of BM sections (*N*) between those two stages can be described by Eq. 7.

7 $$ R_{1} \left( N \right) = R_{1} \left( {N = 1} \right) \cdot N $$

As the result, the energy modulation required by any 4th generation SLS can be greatly reduced, especially for the high harmonic. Particle tracking simulations with the quantum excitation and radiation damping being considered were used to confirm—with an increased number of BM sections up to 10, there exists no degradation of the modulated longitudinal phase space.

## Discussion

The EEHG seeding option has been demonstrated with the capability of generating very narrow bandwidths and extremely high brightness, realized by diffraction-limited short pulses in transverse planes and Fourier-limited bandwidth in the soft X-ray spectrum^1,34–39^. Regarding the 4th generation SLSs, momentum compactions are significantly smaller. To cover the soft X-ray spectrum, we choose the seed-laser wavelength of 250 nm. Furthermore, by separating the stage 1 and stage 2 with a few extra BM sections, the required energy modulation can be reduced, leading to higher repetition rate as well as CR output power. Our particle tracking simulation studies based on the APS-U lattice confirm that with the increased number of BM sections above 10, there exists no degradation of the modulated longitudinal phase space. Also, allowing the two-stage separation tunable could open the door for enabling the pump-probe capability via a novel scheme of twin-pulse seeding the same electron bunch scheme with an adjustable delay in the range of 0.1 to 10 ps. Regarding the twin-pulse-seed scheme, one can flexibly build two different types of pump-probe beamlines, driven by various scientific applications. With the stage 1 in common, regarding the first option, one can construct two independently tunable EEHG beamlines, with a full coverage of the EUV (2.5 to 50 nm) to soft X-ray (1.25 to 2.5 nm) spectrum; for the second option, the pump-probe beamline will share the same stage 2 to generate harmonic two-color pump-probe pulses.

## Data Availability

The datasets generated and analyzed during the current study are not publicly available due to the reason that we want to know who has an interest in our datasets but are available from the corresponding author on reasonable request.
